# Drone-based geospatial prediction modeling identifies *Fasciola hepatica* infection risk in the Cusco Highlands of Peru

**DOI:** 10.1186/s40249-026-01420-1

**Published:** 2026-02-12

**Authors:** Bryan Fernandez-Camacho, Antony Barja, Luis C. Revilla, Rodrigo A. Ore, Jose L. Alccacontor-Muñoz, Maria L. Morales, Melinda B. Tanabe, Gabriel Carrasco-Escobar, Miguel M. Cabada

**Affiliations:** 1 Health Innovation Laboratory, Institute of Tropical Medicine “Alexander Von Humboldt”, Universidad Peruana Cayetano Heredia, Lima, Peru; 2 Cusco Branch – “Alexander von Humboldt” Tropical Medicine Institute, Universidad Peruana Cayetano Heredia, Cusco, Peru; 3 Division of Infectious Disease, Department of Internal Medicine, The University of Texas Medical Branch, Galveston, TX USA

**Keywords:** *Fasciola*, Drone imaging, Geospatial modeling, Prediction, Peru

## Abstract

**Background:**

Fascioliasis is a neglected infectious disease affecting agricultural communities worldwide, with the Peruvian Andes among the most severely affected regions. Identifying fine-scale environmental risk patterns could support targeted surveillance and control. We aimed to develop predictive models of *Fasciola hepatica* infection in humans and sheep using drone-derived environmental indices in a rural Andean community.

**Methods:**

We conducted a cross-sectional study in the Huayllapata community, Cusco, Peru. Demographic, socioeconomic, and georeferenced infection data were collected from households and livestock with fascioliasis diagnosed by stool microscopy. High-resolution multispectral and thermal drone surveys were performed in April 2023 to derive environmental, topographic, and climatic indices. Logistic regression, random forest (RF), XGBoost (XGB), and deep learning models were trained using literature-based or principal component analysis (PCA)-based variable selection strategies. Model performance was evaluated using standard and spatial cross validation approaches. Fine-scale probability surface maps were generated across the study area.

**Results:**

Human fascioliasis prevalence was 21.3% of households, while sheep prevalence reached 80%. Under standard cross validation, RF achieved the best performance for human infection using the literature-based approach (accuracy = 0.89, sensitivity = 0.99, specificity = 0.88), while XGB performed best using the PCA-based approach (accuracy = 0.85, sensitivity = 0.75, specificity = 0.85). For sheep infection, XGB achieved the highest performance (accuracy = 0.93, sensitivity = 0.65, specificity = 0.93) with literature-based variables and RF performed best under the PCA-based approach (accuracy = 0.85, sensitivity = 0.75, specificity = 0.86). Spatial cross-validation reduced accuracy and specificity across models but preserved high sensitivity. Probability maps revealed marked spatial heterogeneity in predicted risk within the community, with shifts in the location and magnitude of risk zones when spatial dependence was accounted for.

**Conclusions:**

In this single Andean community, machine learning models integrating drone-derived environmental, topographic and climatic indices, successfully identified *F. hepatica* infection occurrence in humans and sheep. RF and XGB showed the most robust performance under spatial cross-validation, supporting the feasibility of UAV-based approaches for localized *F. hepatica* risk mapping.

**Graphical abstract:**

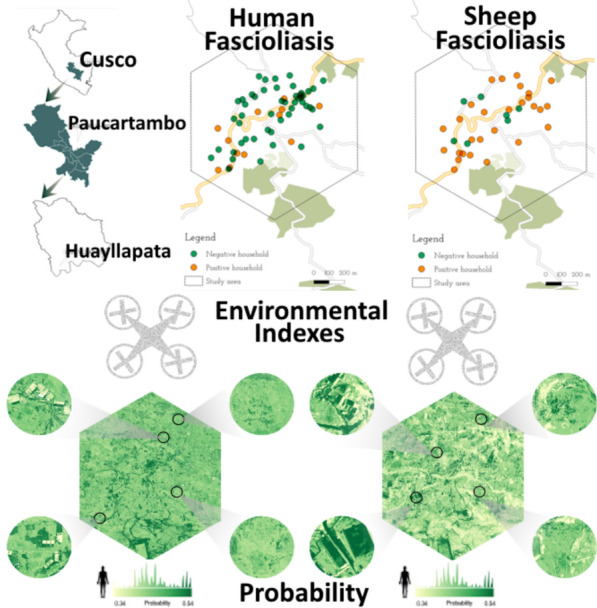

**Supplementary Information:**

The online version contains supplementary material available at 10.1186/s40249-026-01420-1.

## Background

Fascioliasis, a food-borne neglected trematode infection, has a worldwide distribution. Two species cause the disease, *Fasciola hepatica* in Latin America, Asia, Middle East, North Africa and Europe, and *Fasciola gigantica* in Africa and Asia. An estimated 2.4 million people are infected and a large population are at risk in rural communities worldwide [1, 2]. The countries with the highest estimated human burden in South America are Bolivia and Peru where the human prevalence ranges between 15–66% [3]. In Peru, 4500 human cases were reported between 2008 and 2018, most of them occurring in the highlands [4]. A cross-sectional study in the Cusco region found that 10% of children aged 3–16 had a positive test for *F. hepatica*, with infection prevalence varying from 0 to 20% between communities [5]. Children with *Fasciola* exposure were more likely to live at higher altitudes and had lower socioeconomic status, suggesting that the disease is associated with poverty [5].

Fascioliasis is a zoonotic infection occurring in areas where human settlements, livestock farming, and freshwater collections overlap. *Fasciola* infects livestock, lagomorphs, rodents, wild ungulates and humans. The life cycle of fascioliasis involves freshwater snails as intermediate hosts and aquatic vegetation or water as carriers of the infective stage called metacercariae. Environmental factors such as water collection and vegetation, farming practices such as livestock density and irrigation types, and sanitation are critical for the persistence of fascioliasis and makes control highly complex [6]. Modeling to predict the occurrence of fascioliasis in specific geographic locations could provide a powerful tool for strategic control interventions.

Risk maps and predictive models incorporate remote sensing data, such as land cover, temperature, and vegetation indices [7, 8]. Geographic information systems and remote sensing have been used to track areas with high transmission of fascioliasis through the identification of environmental factors associated with infection [9, 10]. Forecasting models for fascioliasis in Brazil and Colombia ranked environmental variables associated with infection incidence to predict risk areas for bovine fascioliasis at the national level [11, 12]. A study in Peru incorporated climatic and vegetation indices derived from satellite imagery to create large-scale risk maps but lacked validation with high resolution data on *Fasciola* distribution among different hosts [13]. This approach is relevant in complex geographic and climatic conditions that make traditional surveillance more difficult [14].

The use of high-resolution images captured by drones offers advanced capabilities for monitoring and detecting infectious agents [15]. Drone-captured images can be used to effectively estimate environmental data, such as soil temperature, humidity, and topography, which are crucial for understanding the transmission of zoonotic diseases [15, 16]. High-resolution multispectral imagery from drones and satellites has demonstrated the feasibility to identify aquatic vegetation associated with snail breeding sites, achieving a segmentation accuracy of up to 94% [17]. By integrating drone data with predictive models, it is possible to identify transmission hot spots and landscape profiles that enhance control strategies across the life cycles of various vectors [18]. In addition, predictive models integrating environmental and socioeconomic data to identify potential transmission hotspots are particularly valuable in regions where conventional surveillance is challenging [19]. This study aims to determine if predictive models using environmental indexes calculated from multispectral and thermal high-resolution images can identify households where humans and livestock are infected with *F. hepatica* in the highlands of Cusco. By comparing these strategies, the study seeks to offer insights into the most effective methods for predicting infection occurrence and guiding intervention efforts. These models have the potential to improve public health responses by enabling more precise and efficient deployment of control measures, ultimately reducing the impact of fascioliasis on vulnerable populations in the Peruvian Andes. Given the modest spatial extent of the study area and the limited number of infected households and animals, our goal was not to derive immediately generalizable risk maps, but rather to evaluate the feasibility of combining unmanned aerial vehicle (UAV)-derived indices with machine learning as a proof-of-concept framework for focal zoonotic disease mapping.

## Methods

### Study area

The Cusco Region is in the southeastern part of Peru and comprises 13 provinces and 112 districts. The total population of the Cusco Region was 1,205,527 in 2017 [20, 21]. The Huancarani district is one of six districts in the Paucartambo province of the Cusco region. The district is at an elevation of 3800 m and had a population of 6911 in 2017, the majority of which lives in rural farming Quechua-speaking communities. Over 95% of the population has Quechua as their native language and 20% is illiterate [20]. Family farming is common practice with most households owning a small area of land and a few sheep, cattle, and/or pigs. Livestock grazing occurs in communal lands and animals are moved throughout the year following the availability of pastures. Huancarani town is located two hours away on a paved road from the Cusco Branch of the Tropical Medicine Institute at Universidad Peruana Cayetano Heredia in Cusco city (CB-TMI).

The climate in the Huancarani District is characteristic of high-elevation Andean environments (~ 3800 m), with significant diurnal temperature variation and seasonal precipitation patterns. Average daytime temperatures typically range between 7–19 °C depending on the season while freezing temperatures may occur at night. The rainy season occurs between November and March, with an average monthly rainfall of 75–150 mm and the dry cold season between April and October, with an average monthly rainfall of 8–45 mm.

### Study design

**Fig. 1:**
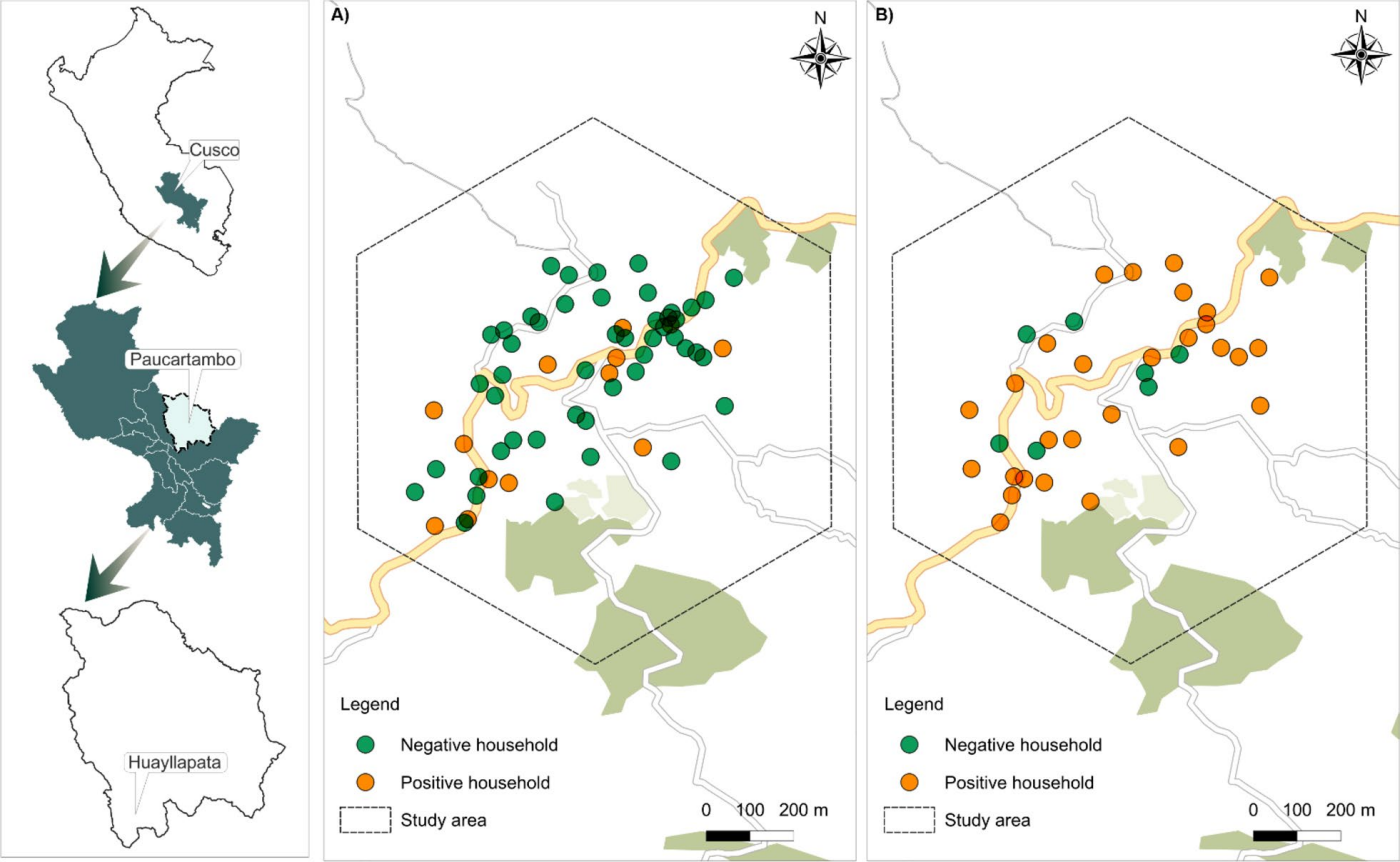
Study area map. **A** Households with human infections, and **B** households with sheep infections. The outlined boundaries indicate the drone flight zone used for mapping

We performed a cross-sectional study in the Huancarani district of Cusco, Peru, as part of a larger One-Health study on fascioliasis in Peru. Farmers in consecutive communities in the district were invited to participate and all children older than three years and adults that provided at least one stool sample and lived in a household with at least one farm animal were eligible to participate. During April 2023, we studied the Huayllapata community and used the information collected there for the present study. Huayllapata (Fig. 1) was selected because previous reports showed a prevalence of *F. hepatica*. between 16–20% [22, 23]. The Huayllapata community spans approximately 1 km^2^, and infections were concentrated in a small subset of households and grazing areas.

### Data collection

#### Epidemiological data

Subjects were enrolled during community meetings and data collection procedures were performed at the subject’s farms. We visited all households where at least one subject consented to participate. During the visits, we collected demographic, socioeconomic, and epidemiologic information using electronic questionnaires created using the Kobo Toolbox platform (Kobo Inc., Cambridge MA, USA). All data was automatically uploaded to the platform through the internet. Geographical positioning system coordinates from the farms were collected using handheld devices (GPS Map 62s, Garmin, Olathe, USA). Coordinates from the front door of the house were used to tag the household and coordinates from the place where stool was collected from livestock to tag the animals. Personnel were trained on data collection procedures using standard operations procedures manuals and retrained when mistakes or inconsistencies were detected. Periodic data audits by the data management team were performed for the detection of inconstancies and their resolution.

We provided each subject a stool collection kit in three separate dates and instructed them to collect a stool sample the next morning. We collected one stool sample directly from the rectum of all livestock owned by the head of the household. All stool samples were preserved at 4 °C in cooler boxes and transported to the laboratory at the CB-TMI for testing within 6–8 h. Lumbreras rapid sedimentation and the Kato Katz microscopy were performed in human stool to detect *F. hepatica* and other helminth eggs or larvae [24]. The Lumbreras rapid sedimentation tests were performed on stool samples preserved in 10% formalin within a week of collection. The Kato Katz tests were performed on fresh human stool within 24 h of collection. Livestock stool samples were tested using a quantitative sedimentation test to detect *F. hepatica* and other helminths eggs or larvae [25]. Audits of laboratory results and retesting of a randomly selected subset of slides or sedimentations by a second observer were performed for internal quality control regularly. External quality control was performed by sending stool sample aliquots to external laboratories performing the same types of tests.

For both human and livestock samples, infection rates were calculated as simple prevalence proportions, defined as:

$$Prevalence \left( \% \right) = \frac{Number\;of\;infected\;individuals}{{Total\;number\;of\;individuals\;examined}} \times 100$$

#### Covariates

We focused our modelling on environmental and remotely sensed covariates (terrain, vegetation, moisture, precipitation, and temperature indices) because these can be measured consistently from UAV and satellite imagery. Household-level socioeconomic and behavioral variables (e.g., sanitation, water source, livestock management, income, education) were not measured in a way suitable to be incorporated in the current models.

#### Drone imagery

High-resolution mapping was performed using thermal and multispectral cameras mounted on drones to characterize the microenvironment composition of the Huayllapata community and calculate environmental and topographic variables. Imaging capture was performed using the Mavic 3 Enterprise Thermal and Mavic 3 Enterprise Multispectral drones. Both cameras captured the Red, Green, and Blue (RGB) bands, while the thermal camera additionally collected the infrared band (IR), and the multispectral camera recorded the red (R), near-infrared (NIR), red edge (RE), and green (G) bands. The RGB bands were captured at wavelengths of 500–570 nm for G, 620–750 nm for R, and 450–495 nm for B. The multispectral bands were captured at wavelengths of 540–580 nm for G, 640–680 nm for R, 710–740 nm for RE, and 770–810 nm for NIR. The thermal band was captured at wavelengths of 8–14 µm (infrared region). The drones were operated from the highest point in the community. The multispectral drone was flown at an altitude of 120 m, resulting in a spatial resolution of 10.5 cm per pixel. The thermal drone was flown at an altitude of 140 m, resulting in a spatial resolution of 33.46 cm per pixel. The flight area covered approximately 1 km^2^, with around 1600 images captured per band by the thermal drone and 1200 images captured per band by the multispectral drone. All images were taken with the camera at an angle set to − 90 degrees and vertical and horizontal overlaps configured at 80%. Drone flights were performed during April 2023.

#### Earth observation data

Climate data, such as precipitation and soil moisture, were obtained from TerraClimate, a global source of high-resolution, gridded monthly climate data. For this study, data for the month of April, the focal period of the investigation, was extracted, covering the years 1958–2023. This extraction aimed to complement the drone-derived environmental and topographic variables (Additional file 1: Table S1) under consideration, providing a comprehensive context for analysis across the 65-year span.

### Data processing

#### Drone imagery processing

The first stage of image processing involved the creation of orthomosaics from drone flights using the Agisoft Metashape v1.5 software (Agisoft LLC, St. Petersburg, Russia). The images were loaded by bands, and alignment was performed based on GPS coordinates and overlapping areas. This process involves detecting key features in overlapping images and matching them, which allows for the calculation of camera positions. Subsequently, tie points were generated, providing the framework for a 3D model. Depth maps were produced for each image, representing the distance from the camera to the surface of the scene, an essential step for generating the dense cloud and digital elevation model (DEM). The dense cloud, which represents a highly detailed 3D model of the surveyed surface, was created from these depth maps. Using the dense cloud data, the DEM was constructed to depict the terrain's elevation. Finally, the orthomosaic was generated as a high-resolution, geometrically corrected 2D map, created by stitching the images together and correcting distortions from terrain or perspective.

The second stage was focused on the generation of environmental and topographic indices from the drone data, associated with the transmission of *F. hepatica* [7, 26–28]. These indices included the normalized difference vegetation index (NDVI), enhanced vegetation index (EVI), soil adjusted vegetation index (SAVI), normalized difference water index (NDWI), soil moisture, slope, aspect, among others (Additional file 1: Table S1). The processing was carried out using the R v.4.4.1 software (R Core Team, Vienna, Austria), where the multispectral bands from each orthomosaic were loaded based on the required indices. The raster data were then aligned, and the relevant calculations for each index were performed.

#### Earth observation data downscaling

Upon acquisition, precipitation and soil moisture data had an initial spatial resolution of 4.6 km per pixel. To enhance the spatial granularity of these datasets, a geographically weighted linear regression model was applied using the *qgisprocess* package [29] in the R software, incorporating NDVI, slope, terrain aspect, and land surface temperature as covariates, which have been previously validated for their effectiveness in estimating climatic conditions [30, 31]. The downscaling process resulted in a refined resolution of 10 cm per pixel, offering a more detailed and accurate representation of the climatic variables across the study area.

#### Modeling

For the predictive modeling of households infected with *F. hepatica*, we applied logistic regression (LR), random forest (RF) and XGBoost (XGB) machine learning algorithms, and a Multilayer Perceptron deep learning (DL) model. All models were implemented in R v.4.4.1, incorporating climatic and environmental indexes obtained from each geo-referenced area at a community level as independent variables. We analyzed the prevalence of *F. hepatica* separately for humans and animals and aggregated prevalence at the household level, serving as two different dependent variables. We selected ovine prevalence for the animal models because this was the most abundant livestock species in the Huayllapata community.

The modeling approach used grid-based prediction of *F. hepatica* infection in households. For this, the study area was divided using a hexagonal grid where predictions were made for each cell based on localized covariates such as precipitation, soil moisture, slope and NDVI (Additional file 1: Table S1). The grid resolution of 50 m in the horizontal and vertical axis for each cell was chosen to ensure accurate spatial representation based on the distribution of the households in the study area. Human and sheep infections were assigned to the hexagon containing the corresponding household or grazing location, and models therefore assumed that exposure was primarily driven by the environmental conditions in that cell.

#### Variable selection

Two strategies were used to select variables for the modeling process: a literature-based approach and a principal component analysis (PCA) based approach. These two strategies were implemented to balance interpretability with the need to reduce dimensionality and collinearity in a setting with a modest number of hexagonal cells. The literature-based selection retained covariates with well-established mechanistic links to *F. hepatica* transmission, which facilitates direct ecological interpretation and translation into field interventions. In contrast, the PCA-based selection compressed highly correlated multispectral and topographic indices into a smaller number of orthogonal components, allowing tree-based and deep learning models to exploit joint variation in vegetation and terrain while limiting the number of effective predictors relative to the sample size.

In the PCA-based approach, 18 variables were grouped according to their primary characteristics: seven topographic, seven vegetation-related, and four general variables (for multispectral bands). Retained principal components accounted for at least 80% of the variance. As a result, two principal components were selected for each of the general, topographic, and vegetation categories, while the wetness variable was excluded from the PCA calculation because it consisted of only one variable.

Under the literature-based approach, Zárate-Rendón et al*.* worked in spatial analysis and risk mapping of *F. hepatica* infection using Slope, NDVI, DEM, precipitation, Land surface temperature (LST) as relevant covariates among dairy cattle in the central highlands of Peru [28].

The PCA-based variable selection consisted in a standardization of the first two components derived from: topographic, vegetation, and general variables, along with wetness.

A total of 16 models were compared to predict households with *Fasciola*-infected humans or sheep.

#### Training and validation

The study area was divided into hexagonal cells and each cell could contain a subset of households. The spatial prediction strategy used the hexagonal area as the study unit where infected and/or non-infected households could be localized. Each household could only be localized in one cell and the outcome label for each cell reflected whether it contained at least one infected household.

Model training and validation were carried out using two stages, using complementary validation strategies. In the first stage, we applied stratified fivefold cross-validation to all four algorithms (LR, RF, XGB and the DL model), ensuring that each fold contained a similar proportion of positive and negative hexagons. This standard cross-validation was used to compare algorithms and to select the best-performing models. In the second stage, to better account for spatial autocorrelation and avoid overly optimistic performance estimates, we implemented an additional spatial cross-validation scheme only for the RF and XGB models, which were chosen as the primary models for risk mapping. In this spatial cross-validation, neighboring hexagons were grouped into three spatial folds, each containing at least one positive hexagon, and entire spatial clusters were held out for testing. For RF and XGB, hyperparameters were tuned using the Optuna framework adapted for R, with up to 1000 trials per model type and predictor set. Hyperparameter optimization was nested within the cross-validation schemes described above to avoid information leakage from the validation folds into the tuning process. Candidate hyperparameters included number of trees, maximum depth, minimum observations per node, learning rate, number of predictors per split, and subsampling ratio. Model discrimination during tuning was evaluated using the receiver operating characteristic-area under the curve (ROC-AUC) metric. All models produced probability maps of *F. hepatica* infection based on the climatic, environmental, and topographic covariates.

A feed-forward deep neural network was developed in R using the Keras library. The network structure consisted of a multilayer perceptron (MLP) with rectified linear unit (ReLU) activation, L2 (ridge) regularization (0.01), and dropout layers (0.10). The final output layer was a single neuron with sigmoid activation. The network was compiled with a binary cross entropy loss function and trained using an Adam optimizer with an exponential decay schedule. Class weights were applied to address class imbalance. The model performance was evaluated for accuracy, sensitivity, and specificity. Training was conducted for 20 epochs with a batch size of eight on an 80/20 train-test split.

#### Surface probability mapping

The predictions generated by each model were spatially represented using a continuous scale of values, ranging 0–1, where higher values indicated a higher likelihood of an infected household. These predictions were estimated for both human and sheep infections within the Huayllapata community area, and were used to visualize environmental suitability at a finer spatial resolution. The resulting raster maps, with a spatial resolution of 10 cm per pixel, provide a high level of detail for localized analysis. All maps were produced using QGIS v.3.40.7 (QGIS Development Team, 2022).

## Results

The analysis of drone-derived indices revealed environmental differences between cells associated with *F. hepatica* infections in humans and sheep. Cells linked to human-infected households had higher median values for the green band (15,081) and red band (10,836) compared to those linked to sheep-infected households with median values of 14,789 and 10,432, respectively. In contrast, NDVI values were slightly lower in human infections (0.16) compared to sheep infections (0.17). NDWI remained consistent across both groups at 0.04 (Table 1).

**Table 1:** Characteristics of negative and positive cells with *Fasciola hepatica* household infections in the Huayllapata community during April 2023

Metrics	Human infection, Median (IQR)		Sheep infection, Median (IQR)	
	Negative, *n* = 419	Positive, *n* = 13	Negative, *n* = 394	Positive, *n* = 28
Green	13,309 (11,761, 15,310)	15,081 (13,591, 17,680)	13,158 (11,638, 15,208)	14,789 (13,563, 17,664)
Red	8599 (7460, 10,491)	10,836 (8751, 12,219)	8467 (7404, 10,182)	10,573 (9270, 12,554)
Red Edge	16,560 (14,788, 18,061)	17,717 (16,100, 18,852)	16,480 (14,786, 18,056)	17,634 (16,577, 18,898)
NIR	13,558 (12,296, 14,880)	13,532 (12,903, 15,368)	13,568 (12,288, 14,944)	13,654 (12,892, 15,368)
LST	15.60 (12.60, 19.80)	17.00 (13.30, 19.80)	15.6 (12.5, 19.8)	16.0 (13.7, 20.3)
DEM	3840 (3798, 3880)	3848 (3826, 3880)	3840 (3793, 3882)	3854 (3826, 3876)
Aspect	115 (95, 137)	109 (102, 132)	115 (95, 137)	122 (102, 146)
CTVI	0.85 (0.80, 0.89)	0.78 (0.76, 0.85)	0.85 (0.81, 0.89)	0.79 (0.74, 0.85)
Curvature	0.001 (− 0.013, 0.012)	− 0.010 (− 0.017, 0.008)	0.001 (− 0.012, 0.012)	− 0.012 (− 0.021, 0.000)
DVI	4761 (2991, 6351)	1838 (1192, 4661)	4814 (3232, 6465)	2301 (766, 5011)
NDVI	0.25 (0.18, 0.32)	0.16 (0.12, 0.26)	0.25 (0.18, 0.32)	0.17 (0.10, 0.26)
NRVI	0.25 (0.18, 0.32)	0.16 (0.12, 0.26)	0.25 (0.18, 0.32)	0.17 (0.10, 0.26)
Precipitation	47.71 (47.33, 48.07)	47.62 (47.36, 47.86)	47.69 (47.31, 48.07)	47.77 (47.47, 47.95)
SAVI	0.38 (0.26, 0.47)	0.24 (0.17, 0.39)	0.38 (0.27, 0.49)	0.25 (0.16, 0.39)
Shade	− 0.03 (− 0.05, − 0.01)	− 0.03 (− 0.05, − 0.02)	− 0.03 (− 0.05, − 0.01)	− 0.03 (− 0.04, 0.00)
Slope	17.30 (14.40, 20.80)	14.30 (13.50, 16.80)	17.5 (14.5, 21.0)	14.5 (13.4, 16.9)
Soil	1040 (1021, 1058)	1033 (1015, 1048)	1039 (1021, 1057)	1042 (1022, 1057)
TTVI	0.85 (0.80, 0.89)	0.78 (0.76, 0.85)	0.85 (0.81, 0.89)	0.79 (0.74, 0.85)
TVI	0.85 (0.80, 0.89)	0.78 (0.76, 0.85)	0.85 (0.81, 0.89)	0.79 (0.74, 0.85)
TWI	6.00 (3.00, 11.00)	3.00 (2.00, 4.00)	6.00 (4.00, 11.00)	3.00 (2.00, 4.00)
Valley Depth	0.28 (0.12, 0.52)	0.27 (0.16, 0.53)	0.26 (0.12, 0.51)	0.34 (0.18, 0.52)
NDWI	− 0.04 (− 0.09, 0.02)	0.04 (− 0.05, 0.07)	− 0.04 (− 0.10, 0.01)	0.04 (− 0.05, 0.08)

*n* Number of grid cells containing positive or negative *F. hepatica* infected households, *NIR* Near infrared, *LST* Land surface temperature, *DEM* Digital elevation model, *CTVI* Corrected transformed ratio vegetation index, *DVI* Difference vegetation index, *NDVI* Normalized difference vegetation index, *SAVI* Soil adjusted vegetation index, *TTVI* Thiam’s transformed vegetation index, *TVI* Transformed vegetation index, *TWI* Topographic wetness index, *NDWI* Normalized difference water index

Topographic and climatic variables showed subtle differences. Slope was slightly lower in cells corresponding to human-infected households (14.3°) compared to sheep-infected ones (14.5°). Shade values remained stable at − 0.03 for both groups. Precipitation and soil values showed minimal variation, with medians of 47.62 mm versus 47.77 mm and 1033 versus 1042, respectively for human and sheep infections.

### Prediction of human infection

**Fig. 2:**
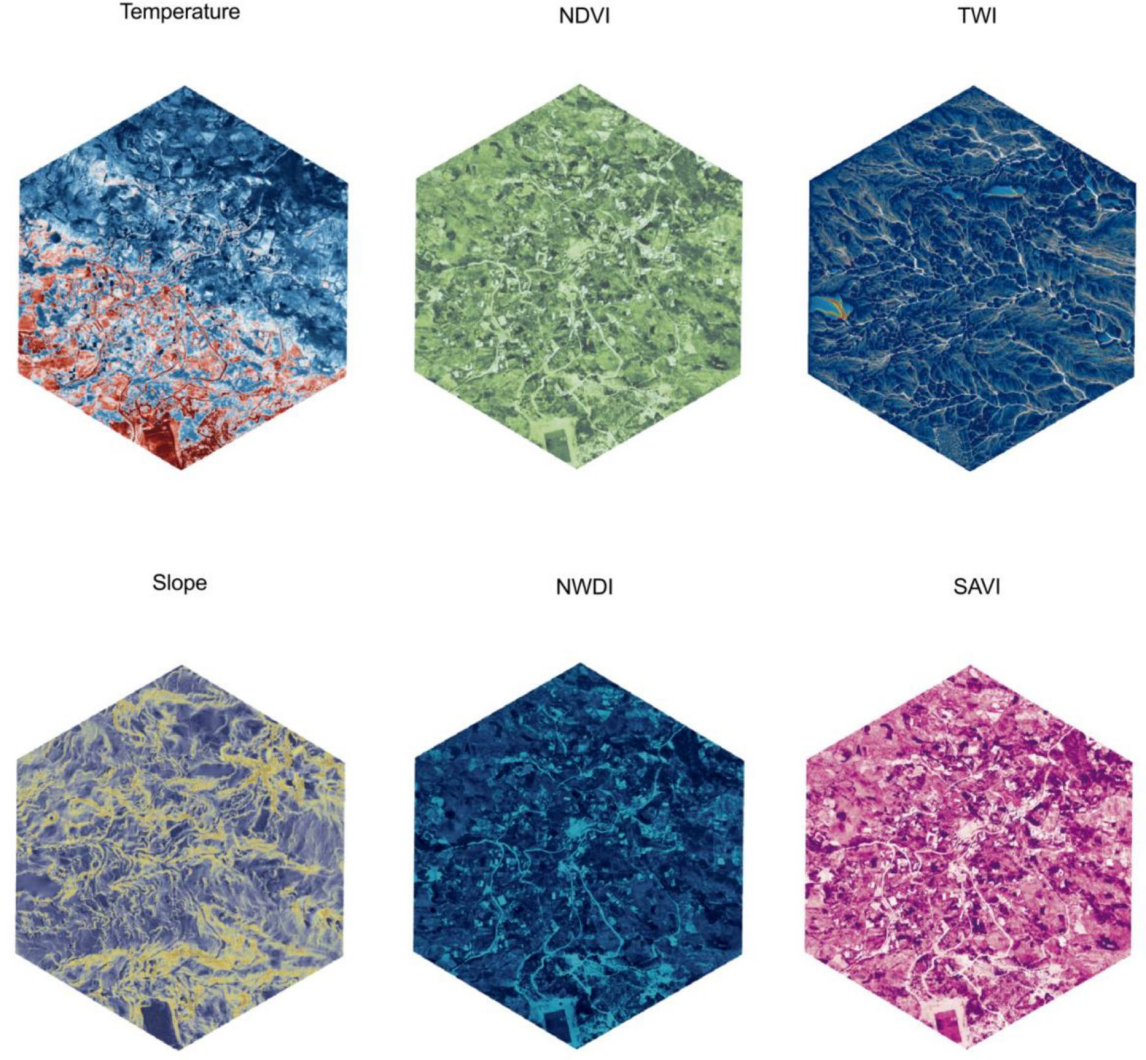
Drone-derived environmental and topographic variables from the Huayllapata community in the Huancarani District. *NDVI* Normalized difference vegetation index, *TWI* Topographic wetness index, *NDWI* Normalized difference water index*, SAVI* Soil adjusted vegetation index

A total of 61 households were included in the human infection prediction analysis. There was at least one human infected with *F. hepatica* in 21.3% of the households (Fig. 1A). The covariates selected for prediction were categorized in topographic, vegetation, water, and general multispectral features for PCA (Additional file 1: Table S1 and visualized in Fig. 2).

#### Standard cross-validation performance

The validation metrics for the eight human infection models under standard fivefold cross-validation are presented in Table 2. Using literature-based variables, RF achieved the best overall performance (accuracy 0.89, sensitivity 0.99, specificity 0.88). XGB showed similar specificity (0.88) but lower sensitivity (0.54, accuracy 0.87), whereas LR yielded moderate and more balanced metrics (accuracy 0.82, sensitivity 0.64, specificity 0.83). Under the PCA-based selection, XGB outperformed the other models (accuracy 0.85, sensitivity 0.75, specificity 0.85), followed by RF (accuracy 0.83, sensitivity 0.60, specificity 0.85) and LR (accuracy 0.58, sensitivity 0.53, specificity 0.58). The DL model achieved very high sensitivity (0.99) at the expense of marked over-prediction of infection (accuracy 0.32, specificity 0.31).

**Table 2:** Performance metrics of predictive models for both human and sheep *Fasciola hepatica* infections in households by variable selection methods under the standard cross validation approach

Infection type	Selection method	Models	Accuracy	Sensitivity	Specificity
Human infection	Literature	LR	0.82	0.64	0.83
		RF	0.89	0.99	0.88
		XGB	0.87	0.54	0.88
		DL	0.77	0.20	0.79
	PCA	LR	0.58	0.53	0.58
		RF	0.83	0.60	0.85
		XGB	0.85	0.75	0.85
		DL	0.32	0.99	0.31
Sheep infection	Literature	LR	0.22	0.97	0.19
		RF	0.88	0.61	0.89
		XGB	0.93	0.65	0.93
		DL	0.52	0.99	0.50
	PCA	LR	0.73	0.62	0.74
		RF	0.85	0.75	0.86
		XGB	0.85	0.68	0.86
		DL	0.10	0.83	0.10

*LR* logistic regression, *RF* random forest, *XGB* XGBoost, *DL* deep learning, *PCA* principal component analysis

#### Spatial cross-validation performance

To account for spatial autocorrelation, we further evaluated RF and XGB using spatial cross-validation on the 2023 data (Table 3). As expected, accuracies and specificities were lower than under standard cross-validation, while sensitivities generally remained high. For the literature-based RF model, accuracy decreased from 0.89 to 0.73 and specificity from 0.88 to 0.72, whereas sensitivity remained at 0.99. The literature-based XGB model showed a similar pattern, with accuracy dropping from 0.87 to 0.69 and specificity from 0.88 to 0.68, but sensitivity increasing from 0.54 to 0.99. For PCA-based models, RF achieved an accuracy of 0.64, sensitivity of 0.60, and specificity of 0.65, while XGB reached an accuracy of 0.70, sensitivity of 0.80, and specificity of 0.71.

**Table 3:** Standard cross-validation and spatial cross-validation metrics comparison for Huayllapata 2023

Model			Cross validation			Spatial cross validation		
			Acc (%)	Sens (%)	Spec (%)	Acc (%)	Sens (%)	Spec (%)
Human	Literature	RF	89	99	88	73	99	72
		XGB	87	54	88	69	99	68
	PCA	RF	84	60	85	64	60	65
		XGB	85	75	85	70	80	71
Sheep	Literature	RF	88	61	89	62	98	61
		XGB	93	65	93	79	99	77
	PCA	RF	85	75	86	76	98	75
		XGB	85	68	86	78	95	77

*Acc* accuracy, *Sens* sensitivity, *Spec* specificity. *RF* random forest, *XGB* XGBoost, *PCA* principal component analysis

**Fig. 3:**
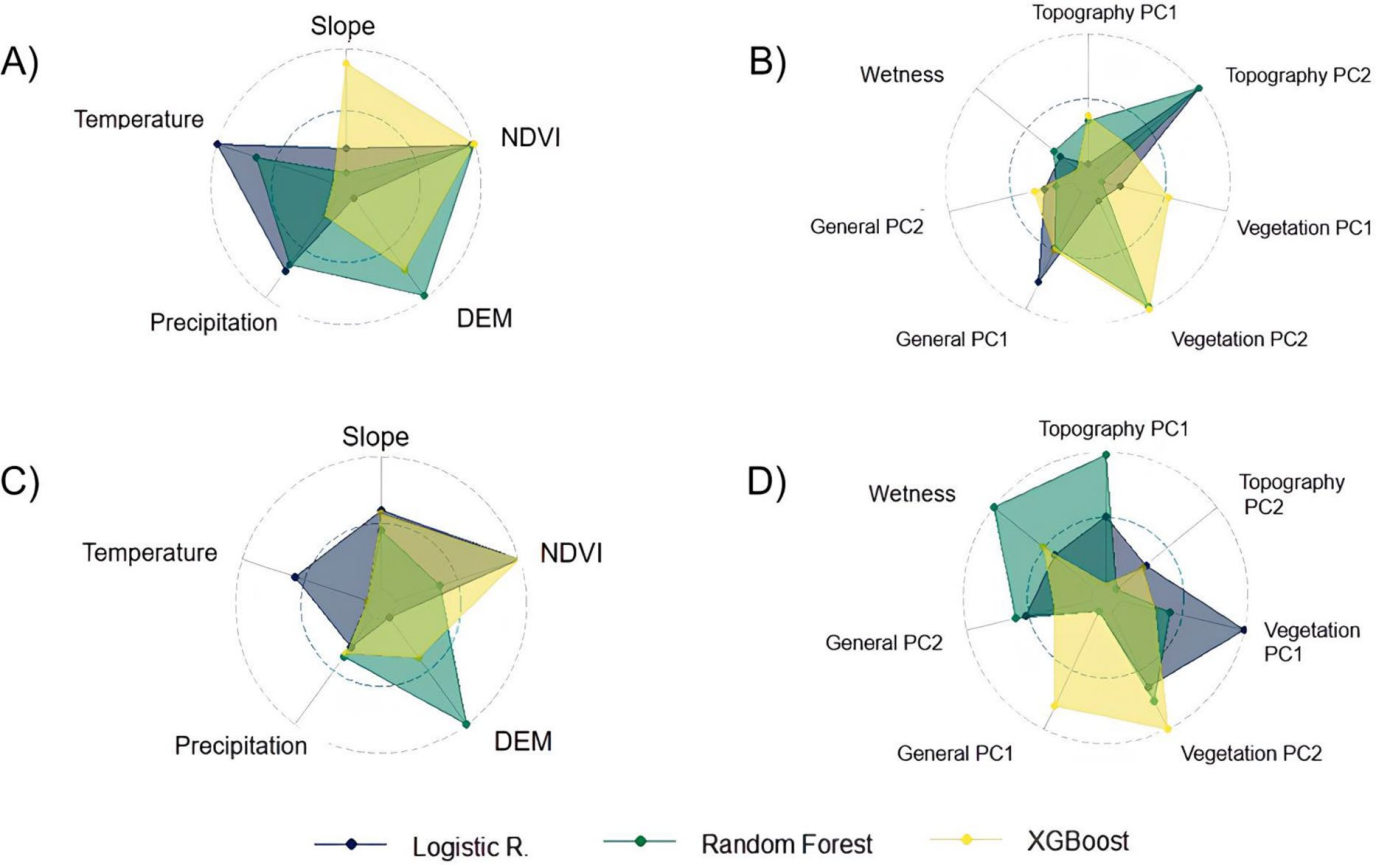
Variable importance under standard cross validation by variable selection method. **A** literature-based selection for human infection, **B** PCA-based selection for human infection, **C** literature-based selection for sheep infection, and **D** PCA-based selection for sheep infection. *NDVI* Normalized difference vegetation index, *DEM* Digital elevation model, *PCA* Principal component analysis

In the literature-based approach, under the standard cross validation (Fig. 3A), temperature, precipitation, NDVI, DEM, and slope were the best predictors. Across the literature-based approach, XGB and RF assigned a higher importance to vegetation indices or their principal components. In the PCA-based approach (Fig. 3B) the topographic and vegetation second principal components were the key contributors.

**Fig. 4:**
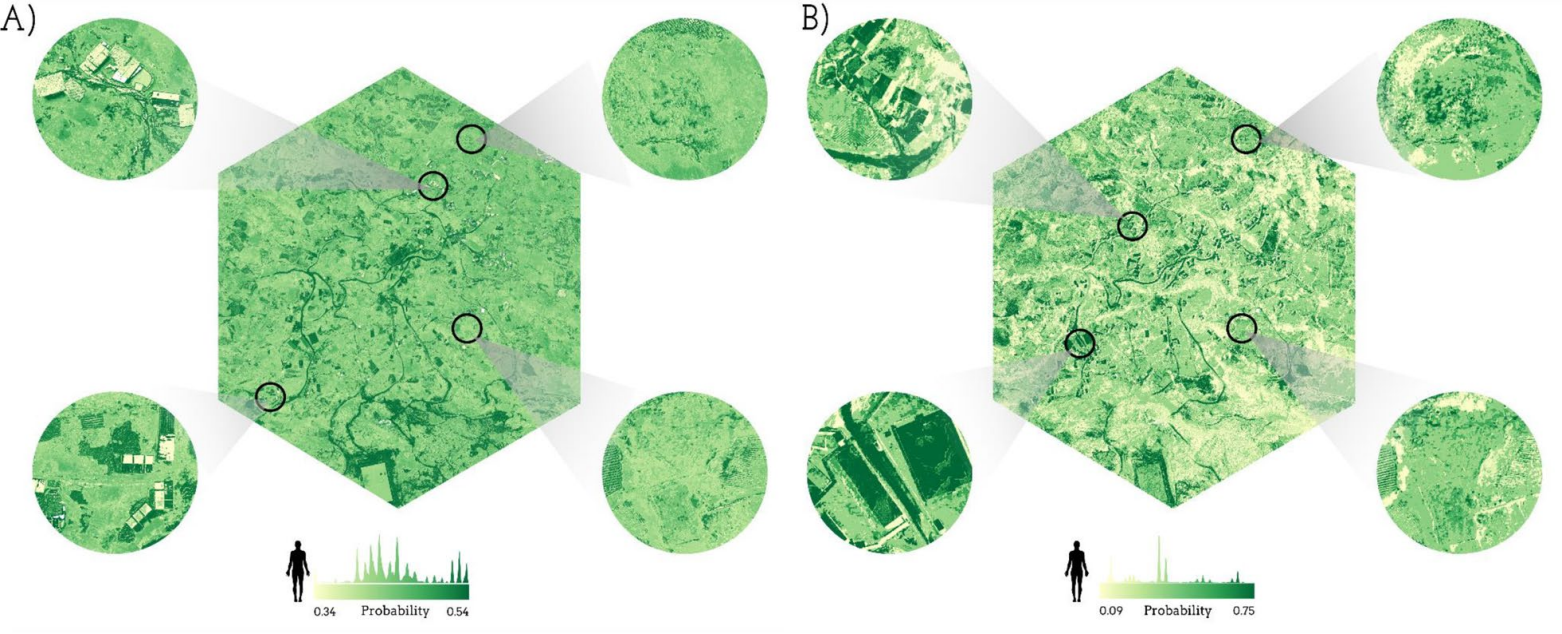
Surface probability maps of *Fasciola hepatica* human infections generated using the PCA-based variable selection method: **A** XGBoost model under standard cross validation and **B** XGBoost model under spatial cross validation. *PCA* principal component analysis

Using the PCA-based variable selection method, the comparison of the surface probability maps, generated with the XGBoost models, shows that incorporating spatial cross-validation substantially alters the magnitude and spatial articulation of predicted infection probabilities across Huayllapata (Fig. 4). In the standard cross-validation model (Fig. 4A), probabilities remain confined to a narrow interval (0.34–0.54), concentrating high probabilities in the central and central-southern areas. However, when spatial structure is introduced (Fig. 4B), the model expands the probability range considerably (0.09–0.75) and produces clearer spatial contrasts. Notably, areas on the central-northern of Huayllapata display marked increases in predicted probability under the spatial model, shifting from mid-range values in the standard model to some of the highest values observed in the entire surface. Conversely, the south-eastern sector, which previously exhibited moderate probabilities, became some of the lowest-probability zones once spatial cross-validation is applied.

### Prediction of sheep infection

In the sheep infection analysis, a total of 35 households were included, with *F. hepatica* detected in 80% of them (Fig. 1B). The validation metrics for eight models predicting *F. hepatica* sheep infections in households are presented in Table 2.

#### Standard cross-validation performance

Table 2 summarizes the performance of the eight sheep infection models under standard fivefold cross-validation. With literature-based variables, XGB achieved the highest overall metrics (accuracy 0.93, sensitivity 0.65, specificity 0.93), followed by RF (accuracy 0.88, sensitivity 0.61, specificity 0.89). LR showed high sensitivity (0.97) but very low specificity (0.19, accuracy 0.22), and DL achieved high sensitivity (0.99) with moderate accuracy (0.52) and low specificity (0.50). Under the PCA-based selection, RF and XGB performed similarly, both with accuracies of 0.85 and specificities of 0.86, and sensitivities of 0.75 and 0.68, respectively. LR again showed more balanced but lower metrics (accuracy 0.73, sensitivity 0.62, specificity 0.74), while DL strongly over-predicted infection (accuracy 0.10, sensitivity 0.83, specificity 0.10).

#### Spatial cross-validation performance

Spatial cross-validation results for the 2023 sheep models (Table 3) confirm that performance is lower when spatial dependence is explicitly accounted for. For literature-based models, RF accuracy decreased from 0.88 to 0.62 and specificity from 0.89 to 0.61, whereas sensitivity increased from 0.61 to 0.98. Literature-based XGB also showed a decline in accuracy (0.92–0.79) and specificity (0.93–0.77) with very high sensitivity (0.99). Under the PCA-based selection, RF achieved an accuracy of 0.76, sensitivity of 0.98, and specificity of 0.75, while XGB obtained an accuracy of 0.78, sensitivity of 0.95, and specificity of 0.77. These spatially validated results suggest that RF and XGB retain good sensitivity for detecting positive hexagons, but with more conservative estimates of accuracy and specificity than indicated by standard cross-validation; we therefore prioritize the spatial cross-validation metrics when assessing model performance for sheep infection.

In the literature-based models (Fig. 3C), the XGB and LR assigned greater importance to vegetation predictors (NDVI), whereas RF assigned the importance to DEM. Under the PCA-based selection (Fig. 3D), vegetation components remained influential for XGB and LR, while RF underscored the role of topographic and wetness variables.

**Fig. 5:**
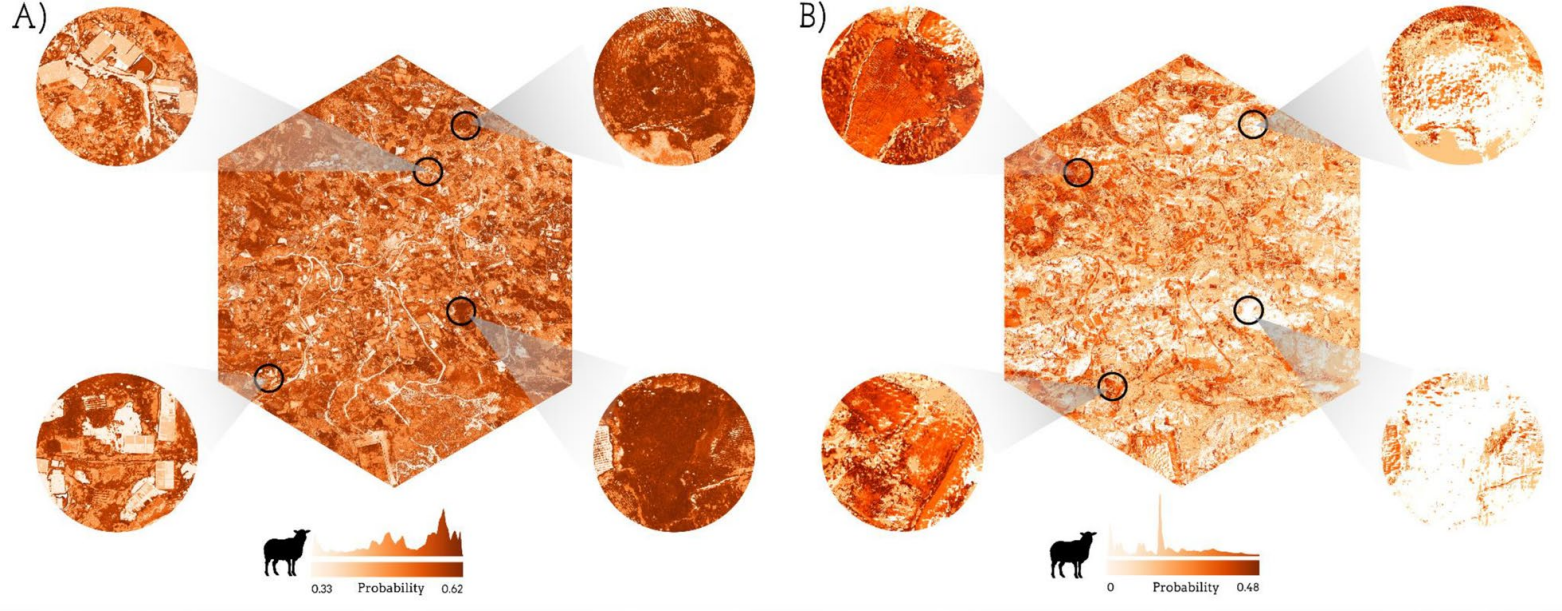
Surface probability maps of *Fasciola hepatica* sheep infections generated using the PCA-based variable selection method: **A** Random forest model under standard cross validation and **B** random forest model under spatial cross validation. *PCA* principal component analysis

Following the PCA-based variable selection method, under the standard cross validation (Fig. 5A), the RF predicted surface probabilities remain within a moderate range (0.33–0.62) and display a coherent spatial structure, with elevated probabilities concentrated mainly in the north-western and south-eastern areas of Huyallapata, while lower probabilities dominate the center area of the community. In contrast, when spatial cross-validation is applied (Fig. 5B), the model yields a wider dynamic, spanning from 0.00 to 0.48, and produces a more fragmented and heterogeneous spatial pattern. Notably, zones that previously held mid-to-high probabilities in panel A shift to markedly lower predicted values in panel B. Additionally, the north and south-eastern areas, which in panel A retains high probabilities, becomes one of the lowest-probability regions under the spatial model.

## Discussion

The present study integrated high-resolution drone-derived topographic and environmental indices, climatic data, and multiple modeling strategies to predict *F. hepatica* infections at the household level in the Peruvian Andes. Our findings underscore the importance of local-scale geospatial indicators, particularly vegetation, water indices, and terrain characteristics, in estimating the probability of infection. The proposed approach captured these environmental drivers effectively, although differences in predictive performance emerged among the machine learning algorithms and modeling strategies. In addition, our results suggested that the infection risk distribution between humans and sheep is different and may have different determinants.

A key ecological insight from the drone-derived indices is the contrast between cells containing households with infection and those without the infection. According to the proposed spatial approach, cells containing households with fascioliasis were associated with higher reflectance values in the green and red bands and consistently lower NDVI values. These patterns may reflect the presence of vegetation types or ground cover conducive to the reproduction of intermediate snail hosts associated with *F. hepatica* transmission [32, 33]. Lower NDVI in cells containing households with the infection might indicate vegetation stress or certain forms of vegetation that thrive in damp conditions essential for snail survival. The NDWI values were slightly increased in cells with infected households compared to cells without them aligning with the notion that small bodies of water or moist terrain play a significant role in supporting the life cycle of *Fasciola* [34]. These findings are consistent with previous studies that have linked waterlogged pastures and particular vegetation structures to higher *F. hepatica* transmission risk [28, 35]. Roessler et al., using a machine learning approach to model fascioliasis risk, suggested that the availability of fresh water and humid environments was one of the strongest predictors of the presence of *Galba truncatula*, the intermediate host in the life cycle of *Fasciola* [35].

The topographic and climatic variables, such as slope, shade, and precipitation were also associated with infection risk [33, 36]. Our results suggest that cells containing households in areas with milder slopes and in areas with slightly less shade values have a higher probability of *F. hepatica* infection. Milder slopes can facilitate water accumulation, creating microhabitats suitable for snail populations, while prolonged shade tends to limit direct solar radiation, influencing vegetation density and soil moisture [37]. Less shade and mild slopes, associated with slower movement of water, are environmental conditions that may also favor the development of *G. truncatula* populations [38]. Although precipitation differences between cells with positive and negative households were small, even marginal variations in rainfall can sustain localized water bodies, essential for snail breeding. Consequently, these findings highlight the synergistic effect of terrain and climate as critical drivers in the spatial heterogeneity of *F. hepatica* transmission [28, 33, 36].

Among the predictive algorithms, XGB consistently demonstrated robust sensitivity, specificity, and accuracy across the variable selection strategies, which if further validated could aid in surveillance of infection risk [39]. The RF in the literature based variable selection and DL in the PCA based variable selection had a sensitivity of 99% for infection in humans suggesting they may serve as potent tools for initial screening in the infection status of households. However, the DL model had low accuracy and specificity, particularly for human infection in the PCA-based scenario, despite achieving very high sensitivity. This pattern is consistent with overfitting in a data-hungry model applied to a limited number of hexagonal cells with imbalanced outcomes. The Multilayer Perceptron architecture, which treats each cell as an independent tabular observation, may also be less suited than tree-based ensemble methods to capturing non-linear interactions in high-dimensional remote-sensing indices under small-sample conditions. Future work should explore architectures that explicitly encode spatial structure [40], stronger regularization and early-stopping schemes, and training on larger multi-community datasets to determine whether deep learning yields consistent gains over simpler ensemble models in this context.

An additional methodological consideration is that apparent model performance depended on the validation strategy. Under standard fivefold cross-validation, RF and XGB achieved high accuracies and specificities for both humans and sheep (approximately 0.85–0.93), whereas these metrics decreased under spatial cross-validation, while sensitivities generally remained high. This behavior is expected when spatial autocorrelation and the non-independence of neighboring hexagons are explicitly taken into account, and indicates that standard cross-validation can yield overly optimistic estimates of discrimination. We therefore consider the spatial cross-validation results for RF and XGB to provide a more conservative and realistic assessment of model performance when extrapolating risk to unsampled areas within the community, and we rely primarily on these spatially validated models when interpreting the infection risk patterns.

Our variable selection strategies, literature-based and PCA-based, determined the pivotal role of topographic, climatic, and vegetation indices in the risk of *Fasciola* transmission [36, 41, 42]. The literature-based models identified temperature, precipitation, NDVI, and slope as important indices. Similarly, a study by Zarate-Rendon et al. using satellite imaging in the central highlands of Peru identified slope, NDWI, NDVI and EVI as significant indices associated with infection in cattle [28]. PCA-based strategy condensed multispectral and topographic dimensions into principal components, confirming the multi-faceted environmental drivers of parasite transmission risks. Importantly, this strategy allowed the machine learning models to process a reduced set of components, mitigating collinearity and overfitting at the cost of reduced direct interpretability of individual indices.

Our complementary sheep-infection analysis with a higher infection prevalence confirmed the risk distribution patterns. Both RF and XGB remained strong performers suggesting that when sheep infection prevalence is high, nuanced local-environmental conditions become more pronounced. Certain modeling approaches may better capture these spatially clustered probabilities.

From a public health and veterinary viewpoint, our findings can be directly applied to the identification of populations at risk of acquiring fascioliasis. Identifying precise geospatial hotspots of *F. hepatica* infection may enable targeted interventions such as improving water drainage, snail habitat disruption, or strategically directed anthelmintic treatment [43]. The demonstrated effectiveness of drone-derived indices in combination with machine-learning approaches underscores the feasibility of using high-resolution remote sensing for disease surveillance in hard-to-reach Andean communities [14]. Important considerations for the adoption of these methods include data availability, costs associated with drone flights, and the complexity of advanced modeling algorithms.

This preliminary study offered insights into *Fasciola* transmission risk but has important limitations that affect generalizability. First, all data were obtained from a single Andean community, and the number of households, particularly those with sheep infection, was modest. As a result, the models may capture local environmental configurations specific to Huayllapata, and performance estimates derived from cross-validation, including spatial cross-validation, should be interpreted cautiously because overfitting cannot be excluded and uncertainty around accuracy, sensitivity and specificity remains substantial. Second, the cross-sectional design precludes assessment of temporal stability in the identified risk patterns, and the drone-based indices represent a single imaging campaign rather than seasonal dynamics. Third, although we included a wide range of environmental and climatic covariates, we did not directly include socioeconomic or behavioral determinants of infection, nor measure some potentially relevant factors such as snail abundance or detailed water management practices at the household level. These factors are likely to play an important role in household-level risk and would be particularly relevant for logistic regression models that explicitly target household infection probability. Our focus on environmental indices reflects a pragmatic first step, leveraging data that can be collected rapidly and uniformly through remote sensing. Fourth, our models implicitly assume static exposure at the household or grazing hexagon, whereas both humans and sheep can move across the landscape and may become infected outside their immediate residence or primary grazing site. The resulting risk surfaces should therefore be interpreted as environmental suitability for infection rather than as precise reconstructions of where infections occurred. Future work should therefore prioritize multi-site validation across different highland settings in Peru and other countries, repeated imaging across seasons, and integration of entomological and behavioral data to confirm the broader applicability of the proposed models and refine their use in routine surveillance.

## Conclusions

This pilot study suggests that integrating drone-captured high-resolution environmental indices with topographic and climatic data and advanced machine-learning models can generate fine-scale maps of environmental suitability for *F. hepatica* infection in a rural Andean community, for both humans and sheep. Within this single-site setting, our models consistently highlighted the importance of vegetation indices such as NDVI, water-related measures, topographic features including slope, and climatic factors such as precipitation and temperature in delineating potential parasite hotspots. Ensemble methods, particularly XGB and RF, emerged as the most reliable approaches under spatial cross-validation, underscoring the advantages of flexible, non-linear models for capturing complex interactions in *F. hepatica* ecology. Although our findings are preliminary and subject to the limitations of scale and sample size, the framework provides a proof of concept that, if validated in larger, multi-site and multi-season studies, could inform targeted interventions with minimal environmental impact and support One Health strategies to reduce transmission and improve public health outcomes in vulnerable Andean communities.

## Supplementary Information

**Additional file 1.**

## Data Availability

The data and codes to perform the data analysis will be available at https://github.com/healthinnovation/tmrc-fasciola/tree/cusco.

## Abbreviations

UAV: Unmanned aerial vehicle
CB-TMI: Cusco Branch of the Tropical Medicine Institute at Universidad Peruana Cayetano Heredia
RGB: Red, green and blue
IR: Infrared
NIR: Near-infrared
RE: Red edge
G: Green
R: Red
nm: Nanometre
µm: Micrometre
cm: Centimetre
km^2^: Square kilometre
GPS: Global positioning system
DEM: Digital elevation model
NDVI: Normalized difference vegetation index
EVI: Enhanced vegetation index
SAVI: Soil adjusted vegetation index
NDWI: Normalized difference water index
LR: Logistic regression
RF: Random forest
XGB: XGBoost
DL: Deep learning
MLP: Multilayer perceptron
PCA: Principal component analysis
LST: Land surface temperature
ROC-AUC: Receiver Operating Characteristic-Area Under the Curve
ReLU: Rectified Linear Unit
L2: Ridge regularization technique
QGIS: Quantum geographic information system
CTVI: Corrected transformed ratio vegetation index
DVI: Difference vegetation index
NDVI: Normalized difference vegetation index
NRVI: Normalized ratio vegetation index
SAVI: Soil adjusted vegetation index
TTVI: Thiam’s transformed vegetation index
TVI: Transformed vegetation index
TWI: Topographic wetness index
NDWI: Normalized difference water index
