# Supplementary material for: Drone-based geospatial prediction modeling identifies Fasciola hepatica infection risk in the Cusco Highlands of Peru
Source: Infect Dis Poverty. 2026 Feb 12;15:22. doi: 10.1186/s40249-026-01420-1 (PMC12895862; doi:10.1186/s40249-026-01420-1)
Supplement: Supplementary file 1 — Additional file 1. [file 40249_2026_1420_MOESM1_ESM.docx]

# SUPPLEMENTARY MATERIAL

Supplementary Tables

Supplementary Table 1. Variables used for the analysis at an initial stage. Topographic, vegetation, water, and general multispectral variables were considered.

| Category | Variable | Description |
| --- | --- | --- |
| General bands | Red band | Reflectance in the red band of the electromagnetic spectrum (620–750 nm). Useful for vegetation analysis. |
| General bands | Green band | Reflectance in the green band of the electromagnetic spectrum (495–570 nm). Helps in analyzing vegetation health. |
| General bands | Red Edge band | Reflectance in the red edge band (710–740 nm), used to monitor chlorophyll content in plants and for vegetation studies. |
| General bands | Near Infrared band (NIR) | Reflectance in the near-infrared band (700–1000 nm). Often used in vegetation indices. |
| Vegetation | Corrected Transformed Ratio Vegetation Index (CTVI) | A vegetation index that improves contrast in dense or sparse vegetation areas. Formula:  $\boldsymbol{CTVI =}\frac{\boldsymbol{(NDVI + 0.5)}}{\boldsymbol{(\vert NDVI + 0.5\vert)}}\boldsymbol{x}\sqrt{\boldsymbol{(\vert NDVI + 0.5\vert)}}$ |
| Vegetation | Difference Vegetation Index (DVI) | Measures the difference in reflectance between the NIR and Red bands. Formula:  $\boldsymbol{DVI = NIR - RED}$ |
| Vegetation | Normalized Difference Vegetation Index (NDVI) | An index used to measure vegetation health. Formula:  $\boldsymbol{NDVI =}\frac{\boldsymbol{NIR - RED}}{\boldsymbol{NIR + RED}}$ |
| Vegetation | Normalized Ratio Vegetation Index (NRVI) | Normalized ratio index useful for identifying green areas. Formula:  $\boldsymbol{NRVI =}\frac{\frac{\boldsymbol{RED}}{\boldsymbol{NIR}}}{\frac{\boldsymbol{RED}}{\boldsymbol{NIR}}\boldsymbol{+1}}$ |
| Vegetation | Soil Adjusted Vegetation Index (SAVI) | Index that minimizes soil influence in areas with low vegetation cover. Formula where L is a correction factor (typically 0.5).  $\boldsymbol{SAVI =}\frac{\boldsymbol{(NIR - RED)}}{\boldsymbol{(NIR + RED + L)}}\boldsymbol{x (1+L)}$ |
| Vegetation | Thiam's Transformed Vegetation Index (TTVI) | A variation of the NDVI transformed. Formula:  *T*$\boldsymbol{TVI =}\sqrt{\boldsymbol{NDVI + 0.5}}$ |
| Vegetation | Transformed Vegetation Index (TVI) | A transformation of the vegetation index to enhance contrast. Formula:  $\boldsymbol{TVI =}\sqrt{\frac{\boldsymbol{(NIR-RED)}}{\boldsymbol{(NIR+RED)}}\boldsymbol{+0.5}}$ |
| Topography | Land Surface Temperature (LST) | Temperature of the Earth's surface measured in degrees Celsius. It is crucial for analyzing energy exchange between the surface and the atmosphere. |
| Topography | Digital Elevation Model (DEM) | Digital elevation model representing the topography of the terrain, indicating the altitude of each point on the Earth's surface. |
| Topography | Slope | The slope of the terrain, measured in degrees or percentage. Calculated from the DEM. |
| Topography | Aspect | The orientation of the slope, expressed in degrees from north (0°–360°). Calculated from the DEM. |
| Topography | Plan Curvature | Describes the curvature of the surface in the direction of water flow. Useful for studying erosion and water accumulation. |
| Topography | Valley Depth | The depth of valleys, measured as the vertical distance between the valley bottom and the highest point on the surrounding edges. |
| Topography | Hillshade | Visual representation of terrain that simulates the effect of sunlight on a surface. Hillshade calculates how light interacts with the terrain’s slope and aspect. |
| Water | Normalized Difference Water Index (NDWI) | Spectral index used to detect water bodies. It highlights the presence of water by enhancing the contrast between water and vegetation or other land surfaces.  $\boldsymbol{NDWI =}\frac{\boldsymbol{(GREEN - NIR)}}{\boldsymbol{(GREEN + NIR)}}$ |
| Water | Topographic Wetness Index (TWI) | Index relating water accumulation and slope to identify potentially wet areas. Formula where $\boldsymbol{\alpha}$ is the contributing area and β is the slope:  $\boldsymbol{TWI = ln(}\frac{\boldsymbol{\alpha}}{\boldsymbol{tan(\beta)}}\boldsymbol{)}$ |
